# Supplementary material for: Converging Role for REEP1/SPG31 in Oxidative Stress
Source: Int J Mol Sci. 2023 Feb 9;24(4):3527. doi: 10.3390/ijms24043527 (PMC9959426; doi:10.3390/ijms24043527)
Supplement: Supplementary file 1 [file ijms-24-03527-s001.zip › Supplementary Videos S1.pptx]

## Slide 1
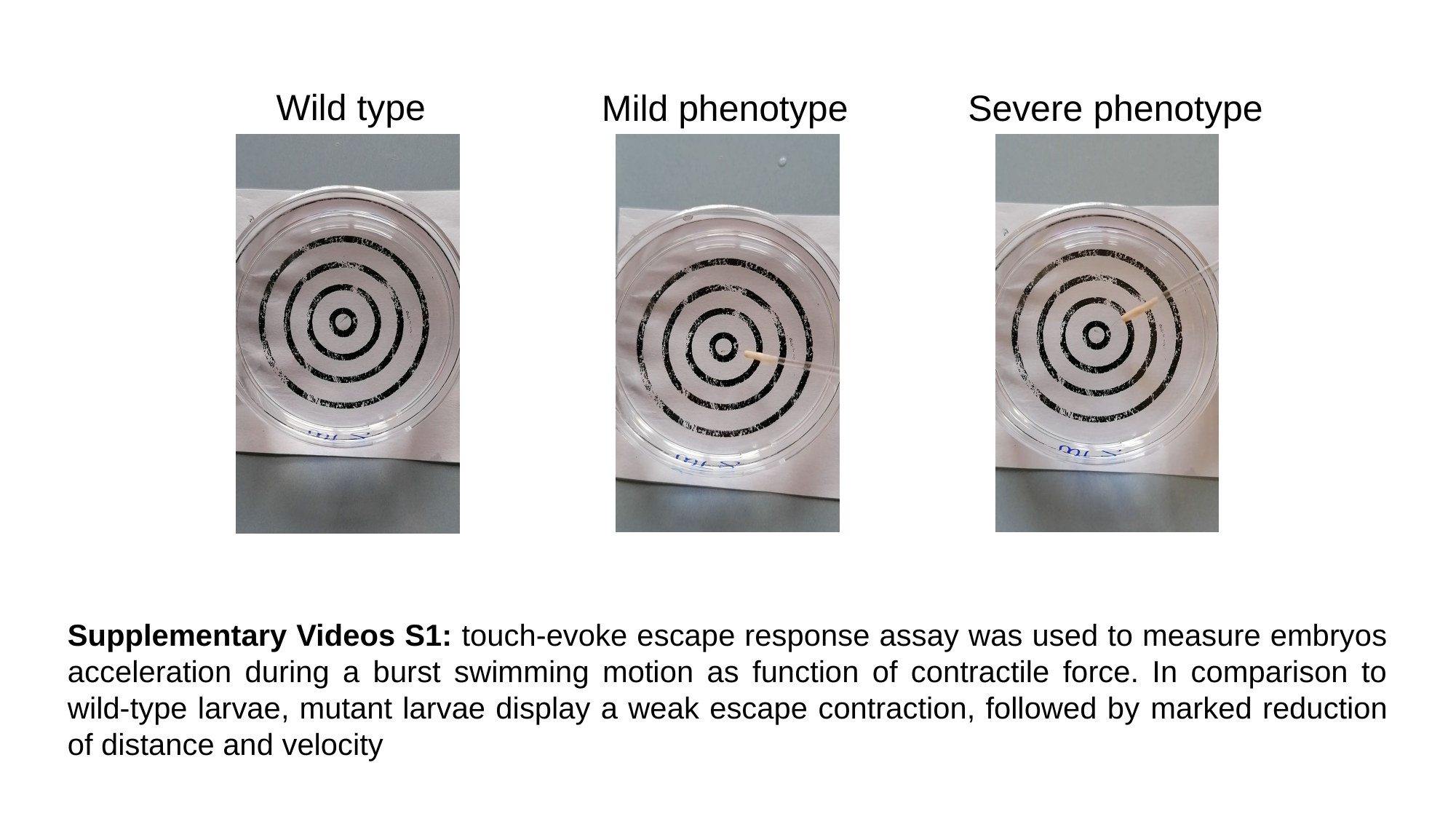

Wild type
Severe phenotype
Mild phenotype
Supplementary Videos S1: touch-evoke escape response assay was used to measure embryos acceleration during a burst swimming motion as function of contractile force. In comparison to wild-type larvae, mutant larvae display a weak escape contraction, followed by marked reduction of distance and velocity
