# Supplementary material for: Converging Role for REEP1/SPG31 in Oxidative Stress
Source: Int J Mol Sci. 2023 Feb 9;24(4):3527. doi: 10.3390/ijms24043527 (PMC9959426; doi:10.3390/ijms24043527)
Supplement: Supplementary file 1 [file ijms-24-03527-s001.zip › Supplementary Table S1_.pdf]

Supplementary Table S1: Clinical and molecular features of SPG31 patients

ID	Sex/Age (years)	Age at onset (years)	SPRS rating scale	ALLELE 1	ALLELE 2	MRI-Cortical	MRI-Basal ganglia	MRI-Cerebellum
Pt1	F/53	23	15/52	duplication (p.?)	WT	-	-	-
P2	M/19	16	6/52	duplication (p.?)	WT	-	-	-
Pt3	F/42	14	11/52	c.337C>T (p.Arg113*)	WT	±	±	-
Pt4	M/38	18	14/52	c.337C>T (p.Arg113*)	WT	-	-	+
