# Supplementary material for: Converging Role for REEP1/SPG31 in Oxidative Stress
Source: Int J Mol Sci. 2023 Feb 9;24(4):3527. doi: 10.3390/ijms24043527 (PMC9959426; doi:10.3390/ijms24043527)

Supplementary Figure S1: steady-state level of the additional proteins involved in mitochondrial dynamics

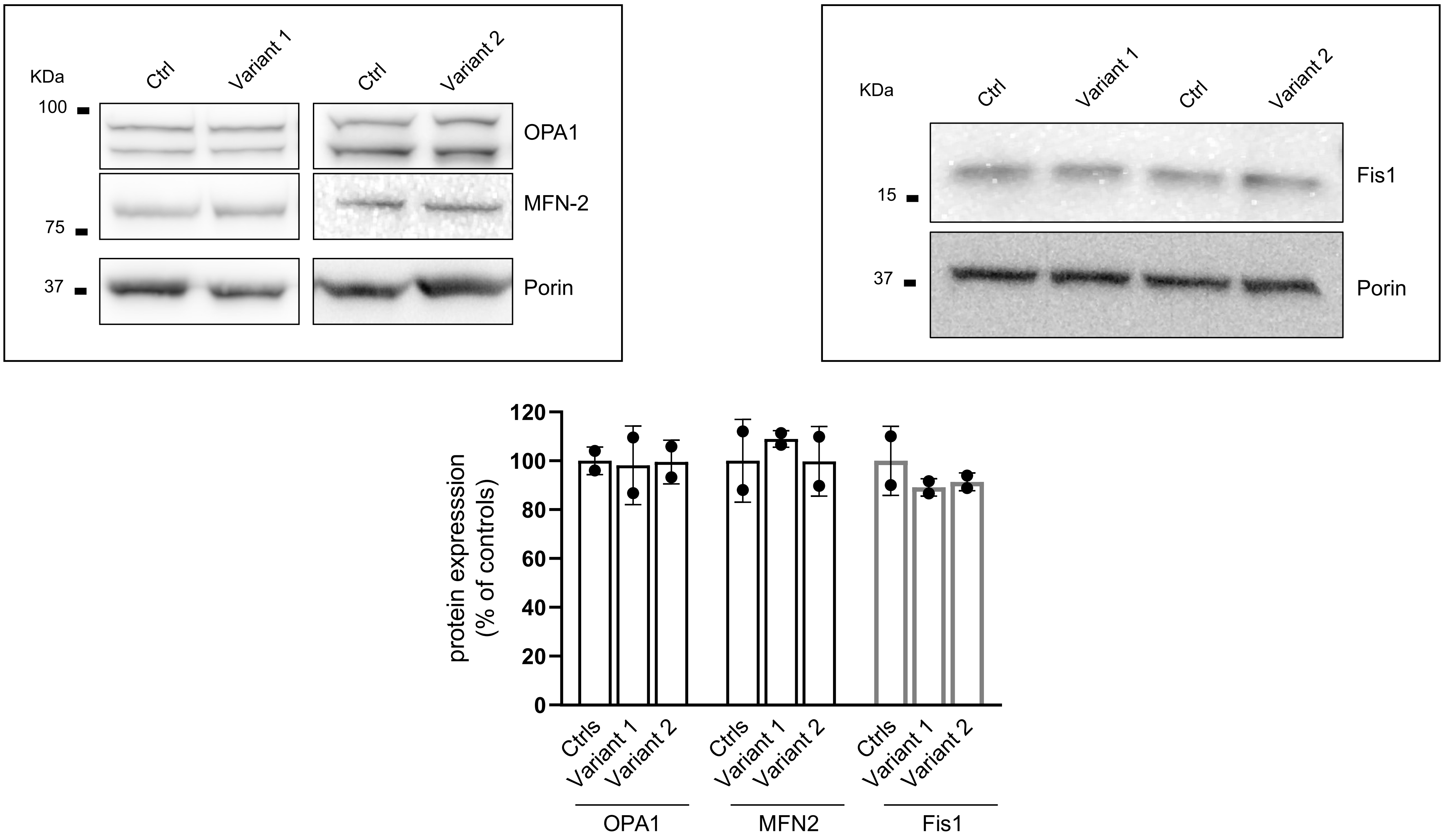

Supplement: Supplementary file 1 [file ijms-24-03527-s001.zip › Supplementary Figure S1.pdf]
