# Supplementary material for: Converging Role for REEP1/SPG31 in Oxidative Stress
Source: Int J Mol Sci. 2023 Feb 9;24(4):3527. doi: 10.3390/ijms24043527 (PMC9959426; doi:10.3390/ijms24043527)
Supplement: Supplementary file 1 [file ijms-24-03527-s001.zip › Supplementary Figure S2.pdf]

Supplementary Figure S2: Multiple alignments of the REEP1 amino acid sequences by Protein BLAST (https://blast.ncbi.nlm.nih.gov/). Protein sequence comparisons reveal the 74% of identity between human (ENST00000538924.7) and zebrafish (ENSDARG00000014854).

```
hREEP1  1    MVSWIISRLVVLIFGTLYPAYYSYKAVKSKDIKEYVKWMMYWIIFALFTTAETFTDIFLCWFPFYYELKIAFVAWLLSPY  80
zreep1  1    MVSWIISRLVVLIFGTLYPAYSSYKAVKSKDVREYVKWMMYWIIFALFTTVEVITDIFLCWLPFYYELKIAFVVWLLSPY  80

hREEP1  81   TKGSSLLYRKFVHPTLSSKEKEIDDCLVQAKDRSYDALVHFGKRGLNVAATAAVMAASKGQGALSERLRSFSMQDLTTIR  160
zreep1  81   TKGSSVLYRKFVHPTLSSKEKDIDEYLCQAKDKSYDTLMHFGRKGLNVAATAAVMAATKGQGVLSERLRSFSMQDLSSFQ  160

hREEP1  161  GDGA--PAPSGPPPPGSGRASGKHGQPKMSRSASESASSSVCTCCSTCRTWKVVEGD--------------VNEGGMKAW  224
zreep1  161  AEGQTNSASSVTTQP----AAAQHRTRTMMRSKSETGYSKGHDFDMTEYELLNLEQSKEPPIPPTPTPILTLSQPNLPQT  236

hREEP1  225  EP-----------HQVQNPLAFSDD--EEEDLL----DFMYKYKAPRRMELPLEAPPRILR--SRFRKKSTSSSATETT  284
zreep1  237  EPVTPLPSPPESQDQSSPPTVASEEPQEKEDFISSSPQFRFKRRAP-------EPPPRPLRPFTRSRSKNALSSDTEAM  308
```
